# Quinoa flour as a skim milk powder replacer in concentrated yogurts: Effect on their physicochemical, technological, and sensory properties

**DOI:** 10.1002/fsn3.2771

**Published:** 2022-02-24

**Authors:** Fatemeh Alkobeisi, Mohammad Javad Varidi, Mehdi Varidi, Majid Nooshkam

**Affiliations:** ^1^ 48440 Department of Food Science and Technology Ferdowsi University of Mashhad Mashhad Iran

**Keywords:** concentrated yogurt, functional food, Quinoa flour, sensory properties, skim milk powder, texture

## Abstract

Milk standardization with solids (i.e., nonfat milk solids, MSNF) for yogurt manufacture is traditionally achieved by the addition of skim milk powder (SMP). However, the addition of SMP to milk‐based yogurt increases lactose content and decreases both protein content and gel firmness. Thus, in this work, quinoa flour (QF; 0%, 25%, 50%, 75%, and 100% w/w) was used to replace SMP in concentrated yogurt. The physicochemical, textural, and sensory properties and microstructure of the yogurt were evaluated during cold storage. Generally, protein content, water‐holding capacity, and L* value decreased, while syneresis, textural attributes, and viscosity increased with increasing QF content. The substitution of high levels of QF (>25%, w/w) for SMP led to significantly shorter fermentation times, as compared to the control sample. The scanning electron microscopy observations showed significant changes in the yogurt microstructure as a consequence of QF replacement. Samples with 25% (w/w) substitution of QF and control had the highest scores in overall acceptance. According to the results, QF could be applied as an interesting raw material for concentrating the milk‐based yogurt at substitution level of 25% (w/w).

## Graphical abstract

In this work, quinoa flour (QF; 0%, 25%, 50%, 75%, and 100% w/w) was used to replace skim milk powder (SMP) in concentrated yogurt.

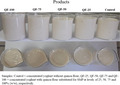

## 1 INTRODUCTION

Concentrated yogurt, a semisolid fermented dairy product (pH ~4.4), is produced by the concentration of milk or yogurt up to 23%–25% total solids (Abu‐Jdayil et al., 2002; Al‐Kadamany et al., 2002; Tamime, 2008). Concentration of yogurt can be performed by mechanical separators after fermentation or by cloth bags (Tamime & Robinson, 2007). Considerable volumes of acid whey are however produced during concentration of the fermented yogurt, which is a major concern for the dairy industry (Jørgensen et al., 2019; Nergiz & Seçkin, 1998). On the other hand, the use of membrane technologies to concentrate yogurt can cause serious problems regarding fouling formation by dairy compounds (Tang et al., 2010).

The concentration of milk‐based yogurt is also performed by the addition of dairy powders (Kashaninejad et al., 2019). Standardization with solids (i.e., nonfat milk solids, MSNF) is traditionally achieved by the addition of skim milk powder (SMP) (Brückner‐Gühmann et al., 2019). However, the addition of SMP to milk‐based yogurt increases lactose content and decreases both protein content and gel firmness (Jørgensen et al., 2019). It is also noteworthy that dairy powders could lead to higher end prices (Uduwerella, 2017).

Over the last few years, plant proteins have been used to improve nutritional value and protein content of various food products because they are rich and inexpensive sources of protein and calories (Akin & Ozcan, 2017). Although yogurt is mainly standardized by dairy powders, several assays have been done to fortify yogurt with oat protein concentrate or isolate (Brückner‐Gühmann et al., 2019), lentil flour (Zare et al., 2011), pea protein isolate, soy protein isolate, and wheat gluten (Akin & Ozcan, 2017) to increase the dry material content of the product.

Quinoa (*Chenopodium quinoa* Willd.) is a dicotyledonous pseudograin with 13%–20% protein and 48%–69% starch contents (Elsohaimy et al., 2015; Li et al., 2016; Sezgin & Sanlier, 2019). The quinoa seeds are rich in essential amino acids with high nutritional value, and the protein quality of quinoa seeds is therefore comparable to that of whole dry milk (Ng et al., 1994). Thanks to its high nutritional value, quinoa (seed/flour) could be therefore introduced as an excellent source to produce functional foods with health‐promoting functions (Curti et al., 2017; Obaroakpo et al., 2020).

To the best of our knowledge, there is no literature report on the use of quinoa flour (QF) as a SMP replacer in concentrated yogurt. Therefore, the objective of this study was to evaluate the impact of replacing SMP by QF on the physicochemical, textural, rheological, and sensory properties of concentrated yogurt, during 21 days of refrigerated storage.

## 2 MATERIALS AND METHODS

### 2.1 Materials

Quinoa seeds were provided from a local market (Mashhad, Iran). Cream (30 g fat in 100 g), milk protein concentrate (MPC; 0.5 g fat, 65 g protein, 7 g ash, and 94.5 g solid nonfat in 100 g), milk (3 g fat, 3.3 g protein, and 8 g solid nonfat in 100 g), and SMP (0.6 g fat, 35.62 g protein, 8.1 g ash, and 95.64 g solid nonfat in 100 g) were provided from Pegah Dairy Co. (Mashhad, Iran). Other chemicals and reagents were purchased from Merck Co. (Darmstadt, Germany).

### 2.2 Flour preparation

Flour preparation was made as described by Herrera et al. (2019) with some modification. In order to remove saponins, the quinoa seeds were soaked in 0.1 M NaCl solution (1:4; quinoa: NaCl solution) and shaken at 170 rpm for 1 h. Then, the seeds were washed with distilled water and air‐dried at 55°C. The dried seeds were milled into flour (60‐mesh size) and the obtained flour (QF; 5.82 g fat, 16.53 g protein, 1.96 g ash, and 94.7 g solid nonfat in 100 g) was stored at 4°C until use.

### 2.3 Concentrated yogurt manufacture

Concentrated yogurt samples were manufactured as described previously (Mehanna et al., 2018; Tamime, 2008) with some modification. The MSNF content of milk was standardized to 15% (w/w) by incorporating with SMP and MPC (6.3%, w/w: 0.7%, w/w; SMP: MPC). This milk was used to produce the control yogurt sample without QF addition. The other samples were standardized by substituting different levels of QF (0%, 25%, 50%, 75%, and 100%, w/w) with SMP. To do this, the powders were dispersed in milk by using a laboratory mixer with a low speed for 15 min, followed by storing the milk overnight at 4°C to allow complete hydration. Subsequently, the fat content of milk samples was standardized to 8% by adding cream. Then, samples were preheated to 50°C and homogenized (IKA T18 basic, Ultra‐Turrax, Germany) at 12,000 rpm for 5 min. The homogenized milks were pasteurized at 90°C for 10 min and subsequently cooled to 42°C in an ice‐cold water bath. The starter culture (YoFlex ^®^ Express 3.0 Chr. Hansen), comprised of *Streptococcus thermophilus* and *Lactobacillus delbrueckii* subsp. *bulgaricus* at a ratio of 1:1, was added to samples and the inoculated samples were then incubated at 43℃ until pH 4.5 was achieved. The samples were finally stored at 4°C until analysis.

### 2.4 Chemical analysis

The total solids (TS), protein, fat, and ash contents of QF and samples were analyzed by the AOAC method (AOAC, 2000). Total carbohydrates were calculated by difference in the sum of moisture, ash, fat, and protein contents.

### 2.5 Acidification trend during yogurt fermentation

Acidification trend in the concentrated yogurts was measured by continuous measurement of pH as a function of acidification during yogurt fermentation, according to the method of Zare et al. (2011).

### 2.6 pH and total titratable acidity

The pH values of the sample were measured with a pH meter (MetrohmAG, Herisau, Switzerland) at 25℃. The total titratable acidity was determined based on a method introduced by Zannini et al. (2018).

### 2.7 Susceptibility to syneresis

The syneresis extent of the samples was monitored after the complete fermentation (i.e., 24 h). Twenty grams of the yogurt was spread on the surface of the filter paper (Whatman filter paper, number 1) placed on a Buchner funnel. In the next step, the funnel was attached to an Erlenmeyer flask which was previously connected to a vacuum pump. The yogurt was then filtered under vacuum for 10 min, and the filtrate was weighed. The syneresis value was then calculated as follows (Supavititpatana et al., 2008):

$$\text{Syneresis}\left(\%\right)=\frac{\text{Filtrate}(g)}{\text{Initial weight of yogurt}(g)}\times 100$$

### 2.8 Water‐holding capacity

Water‐holding capacity (WHC) of the concentrated yogurt sample was determined by the centrifuge method. The yogurt sample (10 g) was added to a tube and then stored at 4℃ for 24 hr. Afterward, the tube was centrifuged at 5000 *g* for 10 min at 4℃. The whey separated from the samples was weighed for WHC measurement (Ilyasoğlu et al., 2015).

### 2.9 Texture analysis

Textural analysis was done by the back extrusion test using a Texture Analyser (TA‐XT Plus, Stable MicroSystems Ltd., UK) following the method of Serra et al. (2009). The samples were filled up to height of 45 mm in a glass cylinder with an inner diameter of 50 mm. A 40‐mm diameter flat cylindrical probe penetrated the sample at a constant speed of 2 mm/s up to 15 mm of the sample depth. The derived texture parameters were firmness (peak positive force; g), consistency (positive area; g.s), and adhesiveness (negative area; g.s).

#### 2.9.1 Apparent viscosity

The apparent viscosities of the samples were determined using a Brookfield rotational viscometer (DV‐II+, Brookfield, Middleboro, MA, USA). A cylindrical container with a radius of 1.945 cm was used to measure the viscosity. The spindle used was RV7 type with radius of 0.1588 cm. The viscosity changes were measured in the range of 0–200 rpm.

#### 2.9.2 Color changes

The color of concentrated yogurts was measured using a Chroma‐meter (CR‐410, Minolta Co. Ltd., Osaka, Japan) according to the method of Ahmadian‐Kouchaksaraei et al. (2014). The device was calibrated using the white plate with L* = 98.14, a* = −0.23, b* = 1.89. The L* (lightness), a* (redness: green [−] to red [+]), and b* (yellowness: blue [−] to yellow [+]) indices of the samples were then measured. Additionally, total color difference (ΔE) of the concentrated yogurts was calculated based on the following equation:

$$\Delta E\;=\;{\left[{\left(\Delta L*\right)}^{2}+\;{\left(\Delta a*\right)}^{2}+\;{\left(\Delta b*\right)}^{2}\right]}^{0.5}$$

#### 2.9.3 Microstructure observations

The microstructure of the yogurts was studied by a scanning electron microscopy (SEM). The samples were prepared as reported by Domagała et al. (2013), with some modification. Briefly, the yogurt was cut into an appropriate section (3 mm × 3 mm × 1 mm) approximately 1 cm below the surface, and the sections were then fixed by glutaraldehyde solution (2.5% w/w, in phosphate buffer at pH 7.4) for 1 day. In the next step, the sections were cut into prisms (1 mm × 1 mm ×3 mm) and dehydrated in a graded ethanol series (30%, 50%, 70%, 90%, and 100%). The prisms were then defatted by chloroform and acetone, and freeze fractured in liquid nitrogen. The fragments were melted in absolute alcohol, critical point dried from carbon dioxide, mounted on SEM stubs, and coated with gold by vacuum evaporation. The microstructure of the samples was finally evaluated by a LEO 1450 VP scanning electron microscope (LEO, Germany).

#### 2.9.4 Sensory evaluation

Sensory analyses of concentrated yogurt samples were evaluated by nine trained panelists using a 5‐point hedonic scale (1: don't like at all; 2: don't like very much; 3: indifferent; 4: like a little; and 5: like a lot). Thirty grams of each sample was presented at room temperature in clear plastic containers. Appearance (color, graininess), bitter taste, aroma and flavor, mouth feeling (smoothness), and overall acceptance of the concentrated yogurt samples were evaluated.

#### 2.9.5 Statistical analysis

Data were analyzed based on a completely randomized design in factorial arrangement with two factors of replacement percentage of QF (five levels) and storage time (four levels) using Minitab software (version 16). All experiments were repeated three times. The significant difference between data means was determined by Tukey test at *p* < .05.

## 3 RESULTS AND DISCUSSION

### 3.1 Chemical composition

The chemical composition of concentrated yogurts is presented in Table 1. There were significant differences between the control sample and supplemented yogurts in the terms of TS, protein, ash, and carbohydrate contents (*p* < .05). The QF‐100 sample showed a higher TS content than other samples (*p* < .05), likely due to the water binding capacity of quinoa starch. Starch is gelatinized during milk pasteurization and it could therefore retain some bound water through preventing water evaporation; thereby leading to an increase in the TS of the QF‐100 sample (El‐Shafei et al., 2020). Protein content of the concentrated yogurt decreased significantly (*p* < .05) as a function of QF substitution (>25%, w/w). This could be likely due to the lower protein content of QF compared to SMP. The ash contents of the QF‐75 and QF‐100 samples were significantly (*p* < .05) lower than the control sample. Despite the fact that the carbohydrate content of the yogurt samples increased by substitution of QF for SMP, these changes were only significant at the 100% substitution level (*p* < .05).

**TABLE 1:** Chemical composition of concentrated yogurt

Sample	Total solids (%)	Protein (%)	Fat (%)	Ash (%)	Carbohydrate (%)
Control	23.22 ± 0.306^b^	6.25 ± 0.09^a^	8.1 ± 0.142^a^	1.220 ± 0.028^a^	7.647 ± 0.518^b^
QF−25	23.12 ± 0.683^b^	5.819 ± 0.213^ab^	8.1 ± 0.141^a^	1.200 ± 0.028^a^	7.992 ± 0.293^ab^
QF−50	23.67 ± 0.424^ab^	5.257 ± 0.217^bc^	8.7 ± 0.707^a^	1.090 ± 0.042^ab^	8.620 ± 0.108^ab^
QF−75	23.20 ± 0.376^b^	4.733 ± 0.05^c^	8.7 ± 0.282^a^	1.010 ± 0.014^bc^	8.756 ± 0.846^ab^
QF−100	25.27 ± 0.565^a^	5.025 ± 0.06^c^	9.3 ± 0.141^a^	0.910 ± 0.042^c^	10.031 ± 0.685^a^

Note

Samples: Control = concentrated yogurt without QF; QF‐25, QF‐50, QF‐75, and QF‐100 = concentrated yogurt with QF substituted for SMP at levels of 25%, 50%, 75%, and 100% (w/w), respectively. Values with different letters are significantly different (*p* < .05).

### 3.2 Acidification trend during yogurt fermentation

**FIGURE 1:**
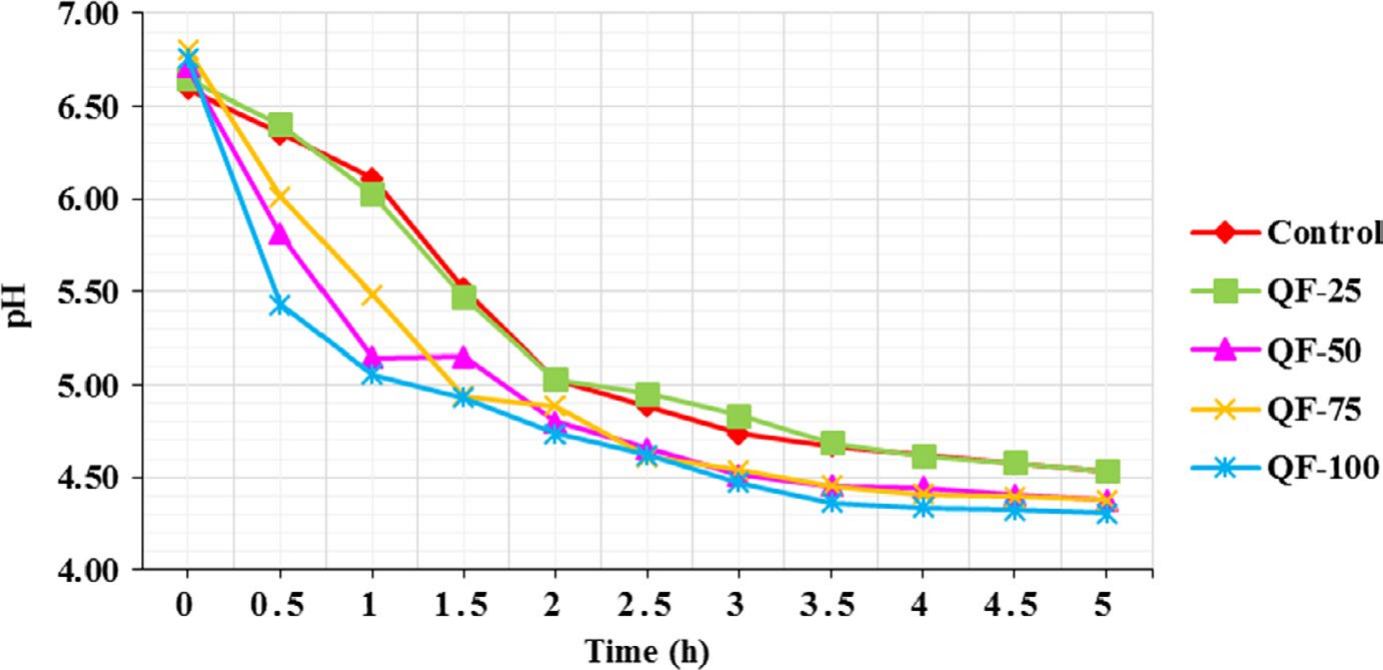
The effect of substitution of QF for SMP on the changes in pH as a function of acidification during fermentation time of concentrated yogurt. Samples: Control = concentrated yogurt without QF; QF‐25, QF‐50, QF‐75, and QF‐100 = concentrated yogurt with QF substituted for SMP at levels of 25%, 50%, 75%, and 100% (w/w), respectively

Acidification trend during yogurt fermentation is shown in Figure 1. The substitution of 100%, 75%, and 50% QF with SMP led to significantly shorter fermentation times, as compared to the control sample (*p* < .05). This effect was significant after 30 min of incubation time for QF‐100 (pH = 5.43) versus control (pH = 6.355) and QF‐50 (pH = 5.81) versus control (pH = 6.355) and after 1 h for QF‐75 (pH = 5.485) versus control (pH = 6.11). The time taken for yogurts to reach pH 4.5 was 3 h for QF‐50, QF‐75, and QF‐100 and 4.5 h for QF‐25 and control. The high protein content of milk‐based yogurts could lead to high buffering capacities and in turn longer fermentation times (Jørgensen et al., 2019). SMP has a high buffering capacity (Brückner‐Gühmann et al., 2019). On the other hand, QF had a lower protein content than SMP. Therefore, a faster increase in acidity of QF‐50, QF‐75, and QF‐100 samples during fermentation may be due to the lower protein content and buffering capacity of the corresponding systems. Another explanation is that the quinoa carbohydrate fraction of the QF‐50, QF‐75, and QF‐100 samples could contribute to this increased acidification rate, since the corresponding samples contained the highest level of carbohydrates. In line with our findings, it has been reported that modifying the carbohydrate composition of milk results in an increase in the acidification rate of yogurt starters (Zare et al., 2012).

### 3.3 Changes in acidity and pH during storage

**FIGURE 2:**
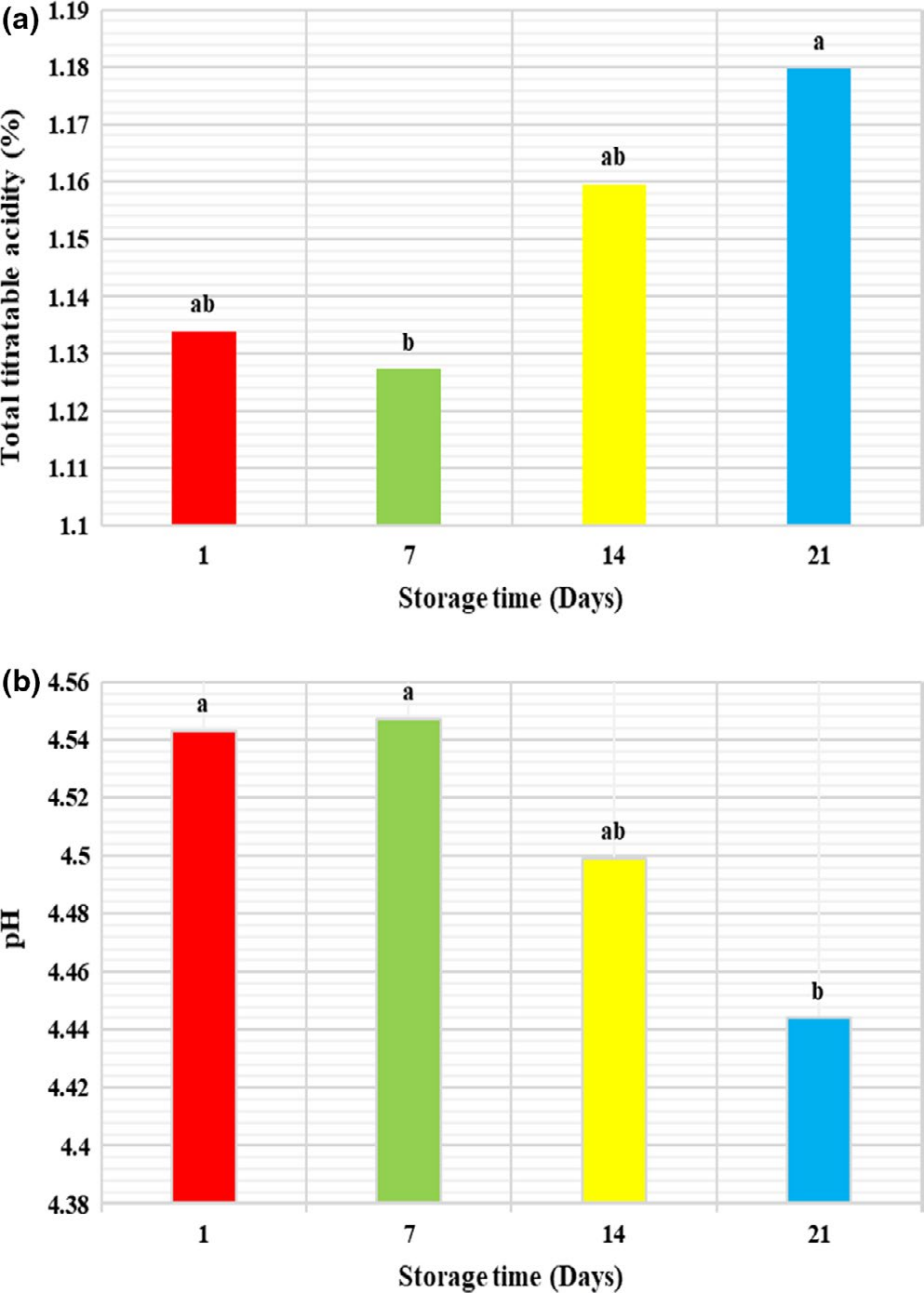
The effect of substitution of QF for SMP on the changes in total titratable acidity (a) and pH (b) of concentrated yogurt during storage. Values with different letters are significantly different (*p* < .05)

The pH value of the yogurt samples was approximately 4.5 after fermentation. In general, the lowest acidity value was measured after 7 days of storage as compared to 1 and 21 days (Figure 2a). The decreased acidity of the yogurts may be due to the proteolysis process and subsequently free amine production during storage time (Alirezalu et al., 2019). The pH value of all yogurt samples declined significantly (*p* < .05) from 4.5 to 4.4 after 21 days of storage period (Figure 2b). This can be due to the utilization of residual lactose by the activity of lactic acid bacteria during storage, that is, postacidification (Casarotti et al., 2014).

### 3.4 Syneresis

**FIGURE 3:**
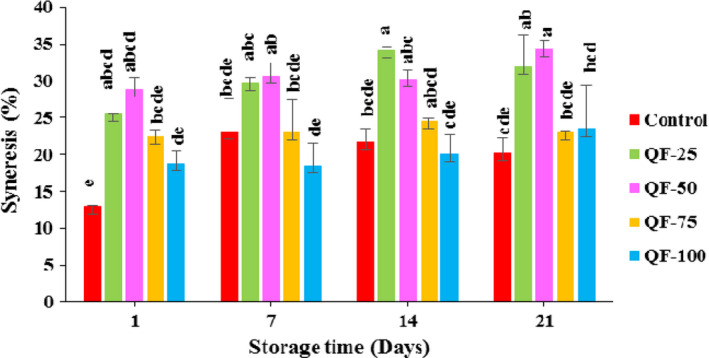
The effect of substitution of QF for SMP on the changes in syneresis of concentrated yogurt during storage. Samples: Control = concentrated yogurt without QF; QF‐25, QF‐50, QF‐75, and QF‐100 = concentrated yogurt with QF substituted for SMP at levels of 25%, 50%, 75%, and 100% (w/w), respectively. Values with different letters are significantly different (*P* < .05)

Figure 3 shows the syneresis value of QF substituted yogurt and control samples. Syneresis occurred due to the weakening of the gel network and in turn the inability of the yogurt gel to retain all of the serum phase (Zoidou et al., 2017). The lowest syneresis value was measured in the control, QF‐100, and QF‐75 samples (*p* > .05); however, the QF‐25 and QF‐50 yogurts had the highest level of whey separation compared to other samples (*p* < .05). The lower syneresis value of the QF‐75 and QF‐100 yogurts could be probably due to their firmer texture (see section 3.6.1). Also, the presence of starch and fiber in QF could reduce free water molecules mainly due to their water binding ability (James, 2009). Syneresis value increased significantly (*p* < .05) with increasing storage time in all samples. This can be related to the casein network rearrangements as a result of pH changes during storage time (de Almeida et al., 2018). Moreover, starch retrogradation in QF‐contained yogurts could enhance the unsightly occurrence of syneresis during storage period (Agyemang et al., 2020). Quinoa starch has 17.1% amylose and 89.9% amylopectin (Araujo‐Farro et al., 2010) with A‐type polymorph and very small polygonal granules (Li & Zhu, 2017; Zhu et al., 2020). The increase in syneresis level during storage period has been ascribed to the interaction between leached amylose and amylopectin chains (Wadchararat et al., 2006).

### 3.5 Water‐holding capacity

Generally, the WHC decreased significantly as a function of substitution of QF for SMP (*p* < .05) (Table 2). Among all samples containing QF, the highest and lowest WHC were found in QF‐100 and QF‐50 samples, respectively (80.51 ± 1.45% versus 71.37 ± 1.24%) (*p* < .05). The high WHC of the control sample compared to other samples is probably due to the protein's type and concentration. Unlike QF, the SMP contains casein proteins with the ability to increase the cross‐linking between the components and provide more capillary forces, holding water in the gel structure (Brückner‐Gühmann et al., 2019), as shown by the SEM micrographs (see section 3.9). Quinoa proteins do not have the ability to form a strong protein network compared to milk proteins (Jeske et al., 2018). However, quinoa protein interaction with milk proteins through electrostatic and heat‐induced hydrophobic and covalent interactions could improve its functional properties (Considine et al., 2011). On the other hand, due to casein content depletion in conjugation with the increase in QF content, it is clear that the increase in WHC in QF‐100 sample could be attributed to the increase of starch ratio in the system and its subsequent gelatinization. During the heating process of milk, quinoa starch gelatinizes and binds water (Ahmed et al., 2018; James, 2009). The lowest WHC in QF‐50 sample may be due to the incompatibility of milk proteins and polysaccharides, which probably led to the formation of large porosity, lower intermolecular cross‐linking, and increased water separation (Corredig et al., 2011; Norton et al., 2015). Such a structure is well visible at 50% replacement, while QF‐100 sample contains a continuous phase rich in starch which covered the protein aggregates (see section 3.9).

**TABLE 2:** The effect of substitution of QF for SMP on the changes in water holding capacity (WHC; %) of concentrated yogurt during storage

Storage (days)	Sample				
	Control	QF−25	QF−50	QF−75	QF−100
1	87.02 ± 0.155^a^	75.07 ± 0.028^efg^	71.13 ± 1.697^g^	78.13 ± 1.053^cde^	81.16 ± 1.619^bcd^
7	84.46 ± 2.291^ab^	77.1 ± 0.579^cde^	71.48 ± 1.414^fg^	77.22 ± 0.098^cde^	80.99 ± 2.107^bcd^
14	82.38 ± 0.445^abc^	78.2 ± 2.213^cde^	71.18 ± 1.817^g^	75.8 ± 0.982^defg^	79.13 ± 1.124^bcde^
21	80.81 ± 1.117^bcd^	76.62 ± 0.424^def^	71.7 ± 1.477^fg^	76.86 ± 0.233^def^	80.55 ± 1.576^bcd^

Note

Samples: Control =concentrated yogurt without QF; QF‐25, QF‐50, QF‐75, and QF‐100 = concentrated yogurt with QF substituted for SMP at levels of 25%, 50%, 75%, and 100% (w/w), respectively. Values with different letters are significantly different (*p* <.05).

### 3.6 Texture profile

#### 3.6.1 Firmness

The QF‐75 and QF‐100 yogurts had significantly higher firmness compared to the control sample (Table 3). This could be likely attributed to the ability of proteins and polysaccharides to form a rigid network through physically‐ or chemically driven interactions, which is generally firmer than the single network structure provided by polysaccharide or protein alone (Lin et al., 2017). Similarly, it has been reported that the firmness of yogurt increased by the addition of 3% and 5% banana flour to the formulation of the flour (Batista et al., 2017). Generally, the firmeness value of all samples was firstly decreased as the storgae time increased from 1 to 7 days, and the parameter was then increased upon further storgae time. It can be explained by the simultaneous decrease of the pH of the samples during storage which makes the gel to shrink (de Almeida et al., 2018). Also, increased firmness in yogurts containing QF during refrigerated storage might occur as a consequence of starch retrogradation, which is associated with the syneresis extent and amylopectin crystallization (Ding et al., 2020).

**TABLE 3:** The effect of substitution of QF for SMP on the texture profile of concentrated yogurt during storage

Parameter	Storage (days)	Sample				
		Control	QF−25	QF−50	QF−75	QF−100
Firmness (g)	1	465.6 ± 59.020^cdef^	376.1 ± 56.864^ef^	511.5 ± 4.475^bcdef^	453.4 ± 19.231^cdef^	844.6 ± 20.982^a^
	7	321.4 ± 7.459^f^	435.8 ± 83.971^def^	334.9 ± 2.735^ef^	543.5 ± 44.430^bcde^	595.5 ± 59.020^bcd^
	14	409.3 ± 20.474^def^	415.6 ± 59.434^def^	381.3 ± 20.225^def^	531.5 ± 119.365^bcdef^	671.1 ± 76.758^abc^
	21	428.7 ± 96.819^def^	514.3 ± 47.497^bcdef^	414.1 ± 13.843^def^	534.0 ± 40.285^bcdef^	692.9 ± 19.977^ab^
Consistency (g.s)	1	3053 ± 460.894^bcde^	2970 ± 127.837^bcde^	3464 ± 56.178^abcd^	2990 ± 44.724^bcde^	4798 ± 162.973^a^
	7	2064 ± 63.447^e^	3346 ± 372.122^bcde^	2211 ± 30.703^de^	3437 ± 237.919^bcd^	3590 ± 48.846^abc^
	14	2672 ± 97.251^cde^	3303 ± 272.575^bcde^	2492 ± 285.233^cde^	3347 ± 614.523^bcde^	3690 ± 907.066^abc^
	21	2586 ± 498.878^cde^	3473 ± 256.843^abcd^	2703 ± 167.828^cde^	3360 ± 114.999^bcde^	4105 ± 152.755^ab^
Adhesiveness (g.s)	1	−837 ± 123.006^abcd^	−571 ± 20.499^a^	−1056 ± 71.854^de^	−707 ± 16.869^abcd^	−1330 ± 70.448^e^
	7	−595 ± 39.534^a^	−714 ± 73.387^abcd^	−603 ± 37.908^a^	−814 ± 77.490^abcd^	−877 ± 51.545^abcd^
	14	−640 ± 25.402^ab^	−705 ± 56.587^abcd^	−642 ± 80.434^ab^	−760 ± 62.722^abcd^	−968 ± 285.436^bcd^
	21	−648 ± 90.321^ab^	−708 ± 66.212^abcd^	−696 ± 28.312^abc^	−789 ± 27.491^abcd^	−1017 ± 58.192^cde^

Note

Samples: Control = concentrated yogurt without QF; QF‐25, QF‐50, QF‐75, and QF‐100 = concentrated yogurt with QF substituted for SMP at levels of 25%, 50%, 75%, and 100% (w/w), respectively. Values with different letters are significantly different (*p* < .05).

#### 3.6.2 Consistency

Substitution of QF for SMP significantly (*p* < .05) increased the consistency of the concentrated yogurt, except at the substitution level of 50% (w/w) (Table 3). Protein content and longer fermentation time are among the factors that increase the consistency of yogurt (Pakseresht et al., 2017). However, the control sample with higher protein content and longer incubation time had the lowest consistency. The increased consistency in QF‐loaded samples could be probably ascribed to their higher firmness values (Sekhavatizadeh et al., 2021). Also, during milk thermal processing, quinoa starch gelatinizes and absorbs water which leads to an increase in viscosity of the aqueous phase and in turn the effective concentration of milk protein, giving a high consistency (Agyemang et al., 2020; Oh et al., 2007). The storage time had a significant main effect on the consistency of yogurt (*p* < .05). On day 1, all samples had the highest consistency and after 7 days storage, the consistency decreased significantly compared to the first day. It is reported that acidity increment and rearrangement of the protein network during storage increase the consistency of yogurt (Guénard‐Lampron et al., 2020; Katsiari et al., 2002). Therefore, a significant decrease in acidity on the seventh day probably led to a decrease in the consistency of all samples. After 7 days, the acidity of the yogurts was increased and the consistency index was increased, as well.

#### 3.6.3 Adhesiveness

The substituted 100% (w/w) QF for SMP significantly (*p* < .05) increased the adhesiveness, as compared to other samples (Table 3). Adhesiveness is generally related to the starch concentration and gelatinization (Liu et al., 2020). It has been demonstrated that the addition of all types of starches to the yogurt formulation could effectively increase the adhesiveness of the final product (Pang et al., 2019). Therefore, the highest adhesiveness of the QF‐100 sample could be likely due to its higher starch levels than other samples. The storage time had also a significant effect on the yogurt adhesiveness (*p* < .05). The highest adhesiveness of concentrated yogurts (*p* < .05) was observed on the first day. The decrease in adhesiveness during storage time may be due to the increased syneresis value of the samples. In congruence with our results, Pang et al. (2015) reported that the adhesiveness of acid milk gels increased significantly by the addition of higher concentrations of tapioca starch.

### 3.7 Apparent viscosity

**FIGURE 4:**
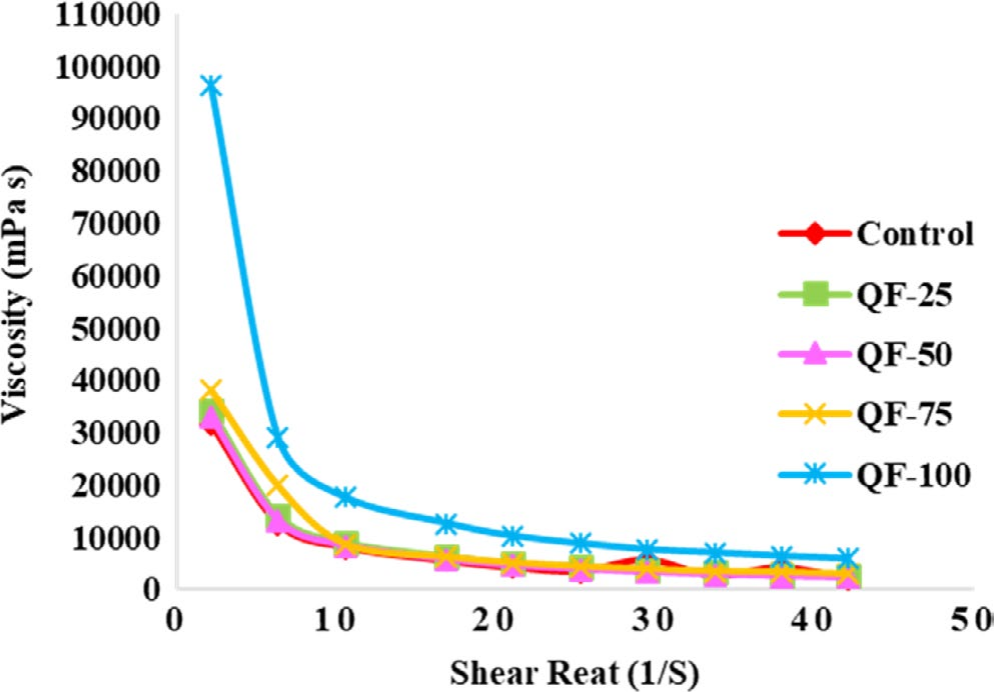
The effect of substitution of QF for SMP on the viscosity of concentrated yogurt. Samples: Control =concentrated yogurt without QF; QF‐25, QF‐50, QF‐75, and QF‐100 = concentrated yogurt with QF substituted for SMP at levels of 25%, 50%, 75%, and 100% (w/w), respectively

**FIGURE 5:**
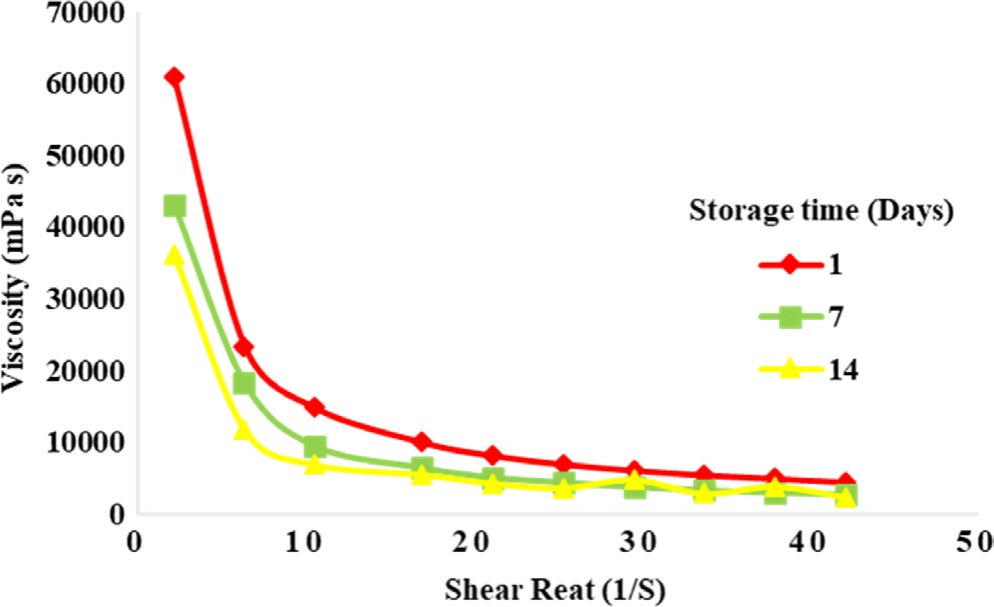
The effect of storage time on the viscosity of concentrated yogurt. Samples: Control = concentrated yogurt without QF; QF‐25, QF‐50, QF‐75, and QF‐100 = concentrated yogurt with QF substituted for SMP at levels of 25%, 50%, 75%, and 100% (w/w), respectively

The apparent viscosity of QF‐100 was dramatically higher (*p* < .05) than that of other samples, probably due to its higher TS and carbohydrate contents (Figure 4). The viscosity increased as a function of starch level in the yogurt formulation because starch granules have the potential to form a stronger protein network through dispersing and filling the gel network. Moreover, starch increases the continuous phase viscosity via amylose solubilization, water absorption (during swelling), and rising protein concentration within the continuous phase (Agyemang et al., 2020). These results were consistent with the adhesiveness observations, which is also considered as an indicator of viscosity (Anbarani et al., 2021). Increase in viscosity, due to the addition of 1% QF to yogurt, has been reported by Codină et al. (2016). The apparent viscosity of all the samples was decreased as the shear rate increased. This can be due to the fact that the applied shear force could break the casein strands and degrade weak electrostatic and hydrophobic interactions in the yogurt network. Therefore, all samples showed a shear‐thinning behavior with shear rate. Other studies reported a similar behavior in concentrated yogurts (Abu‐Jdayil et al., 2002; Costa et al., 2019; Mohameed et al., 2004). The viscosity of all yogurt samples decreased significantly (*p* < .05) compared to the first day (Figure 5). This may be due to weakening of the protein network by proteolytic enzymes secreted from starter cultures in yogurt (Vieira et al., 2019) and the acidic pH of the final product (Demirci et al., 2018).

### 3.8 Color

The color values *L* ^*^, a^*^, and *b* ^*^ of concentrated yogurts are shown in Table 4. All QF substituted yogurts had significantly (*p* < .05) less *L* ^*^ value in comparison to control sample. The highest *L* ^*^ value in SMP arose from the light scattering effect of milk protein and fat particles (Chudy et al., 2020). QF had a lower degree of brightness (*L* ^*^ = 82.08 ± 0.424) than SMP (*L* ^*^ = 99.67 ± 0.197). The brightness of all samples was reduced during the storage time. This could be probably ascribed to the changes in acidity and pH which could lead to calcium phosphate solubilization, casein micelles dissociation, and subsequently micelle size reduction; so, the brightness of sample decreases (García‐Pérez et al., 2005).

**TABLE 4:** The effect of substitution of QF for SMP on the color parameters of concentrated yogurt during storage

Parameters	Storage (days)	Sample				
		Control	QF−25	QF−50	QF−75	QF−100
L^*^	1	94.25 ± 1.269^a^	91.77 ± 1.329^a^	87.31 ± 0.806^bc^	87.12 ± 0.512^bc^	84.09 ± 0.526 cd
	7	91.91 ± 1.721^a^	91.30 ± 0.070^ab^	85.71 ± 0.756 cd	85.35 ± 0.346 cd	81.93 ± 0.162^d^
	14	93.36 ± 2.450^a^	91.60 ± 0.240^a^	85.53 ± 0.604 cd	85.99 ± 2.124 cd	82.60 ± 0.548^d^
	21	93.62 ± 0.830^a^	91.25 ± 0.321^ab^	85.89 ± 0.626 cd	85.82 ± 1.258 cd	82.17 ± 0.014^d^
a^*^	1	−0.530 ± 0.035 cd	−1.158 ± 0.116^e^	0.145 ± 0.035^ab^	0.425 ± 0.530^ab^	0.620 ± 0.035^a^
	7	−0.940 ± 0.240^de^	−1.343 ± 0.017^e^	−0.052 ± 0.017^bc^	0.115 ± 0.007^ab^	0.455 ± 0.127^ab^
	14	−0.863 ± 0.166^de^	−1.303 ± 0.010^e^	−0.062 ± 0.017^bc^	0.150 ± 0.028^ab^	0.361 ± 0.210^ab^
	21	−1.007 ± 0.074^de^	−1.300 ± 0.056^e^	0.015 ± 0.056^abc^	0.025 ± 0.007^abc^	0.515 ± 0.035^ab^
b^*^	1	13.81 ± 0.243^ab^	11.94 ± 0.038^c^	13.51 ± 0.017^ab^	13.23 ± 0.459^b^	14.35 ± 0.014^a^
	7	13.07 ± 0.487^ab^	12.04 ± 0.045^c^	13.18 ± 0.017^b^	13.54 ± 0.162^ab^	13.69 ± 0.286^ab^
	14	13.37 ± 0.205^b^	11.86 ± 0.038^c^	13.11 ± 0.045^b^	13.21 ± 0.183^b^	13.34 ± 0.045^b^
	21	13.85 ± 0.053^b^	11.83 ± 0.113^c^	13.41 ± 0.091^b^	13.42 ± 0.410^b^	13.34 ± 0.109^b^
Δ*E* ^*^	1	–	3.169 ± 0.103^cdef^	6.978 ± 0.471^abcd^	7.241 ± 0.725^abcd^	10.239 ± 1.759^ab^
	7	–	1.718 ± 0.895^ef^	6.280 ± 2.469^bcde^	6.660 ± 1.369^abcde^	10.096 ± 1.543^ab^
	14	–	2.571 ± 1.678^def^	7.875 ± 1.823^abc^	7.442 ± 0.296^abcd^	10.837 ± 2.933^ab^
	21	–	3.174 ± 0.832^cdef^	7.808 ± 0.187^abc^	7.881 ± 0.451^abc^	11.561 ± 0.829^a^

Note

Samples: Control = concentrated yogurt without QF; QF‐25, QF‐50, QF‐75, and QF‐100 = concentrated yogurt with QF substituted for SMP at levels of 25%, 50%, 75%, and 100% (w/w), respectively. Values with different letters are significantly different (*p* < .05).

The QF‐25 and control samples had negative a^*^ values, mainly due to their high levels of SMP and in turn riboflavin pigment, causing a greenish product (Chudy et al., 2020; Zhou et al., 2021). At higher substitutions of QF in yogurt, a^*^ showed positive values that may be related to the increased betacyanin ratio in the samples (Escribano et al., 2017). There was a decrease in the a^*^ values of all yogurts as storage time increased. This is probably due to the increased whey separation during storage, which contains riboflavin (García‐Pérez et al., 2005).

The *b* ^*^ values increased with increasing QF level in the yogurt samples. The QF‐100 and control yogurts had the highest *b* ^*^ values (*p* > .05). The bright‐yellow color of the SMP and the presence of betaxanthins in QF could result in an increase in the *b* ^*^ values of samples (Escribano et al., 2017). After 14 days storage, *b* ^*^ values in QF‐100 yogurt was significantly decreased (*p* <.05). This may be related to the oxidation of the betaxanthin by hydrogen peroxide produced by cultures during storage (Azeredo, 2009; Dave & Shah, 1997). Moreover, the ∆E values of the samples increased significantly (*p* < .05) with increasing QF concentration in the formulation. The overall color changes of substituted samples compared to the control were distinct in QF‐25 (1.5<ΔE<3) and very distinct in QF‐50, QF‐75, and QF‐100 yogurts (ΔE>3). The original color of the SMP was L*:99.67 ± 0.19, a*: −4.63 ± 0.45, and b*:13.87 ± 1.43; however, the QF had color indices of L*:82.08 ± 0.42, a*: −0.16 ± 0.017, and b*:12.54 ± 0.06. The yogurt color is therefore affected by the color difference of the original ingredients.

### 3.9 Microstructure

**FIGURE 6:**
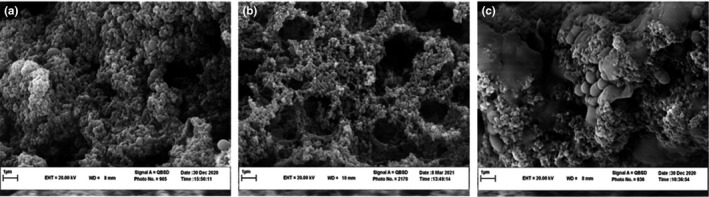
The effect of substitution of QF for SMP on the microstructure of concentrated yogurt. Samples: a = concentrated yogurt without QF; b and c = concentrated yogurt with QF substituted for SMP at levels of 50% and 100% (w/w)

As can be seen in Figure 6a, the microstructure of control yogurt was characterized by a network of chains and clusters of casein micelles. Interaction between clusters and casein chains resulted in numerous small pores which are distributed throughout the network. A similar structure has been reported for concentrated yogurt in other studies (Aly et al., 2020; Ozer et al., 1999). It seemed that substitution of 50% (w/w) QF for SMP (QF‐50) resulted in a weaker network structure with thin casein strands and larger pores (Figure 6b). This structure may be explained by a shorter incubation time, which could result in an accelerated release of colloidal calcium phosphates from casein micelles; indeed, the early release of individual caseins from the micelles is induced by a faster acidification rate and the early development of the casein network is therefore facilitated. This leads to a fast protein aggregation, and a small number of protein–protein bonds could be subsequently formed, which along with extensive rearrangement of the particles/clusters, a weak gel with large pores, and greater whey separation could be resulted. The highest syneresis extent in this sample also confirmed the weak network of the product (Sah et al., 2016).

It was observed that substitution of 100% (w/w) QF for SMP (QF‐100) led to a more compact and strong gel structure (Figure 6c). This may be related to carbohydrate ratio increase in the formula. Proteins are able to form bonds with starch molecules through hydrophilic groups (Goel et al., 1999). Starch acts as a filler compound in protein matrix and leads to an increased gel strength, which is consistent with the results of texture and viscosity analysis (Diamantino et al., 2019; Lin et al., 2017). Furthermore, in some areas, casein clusters and network pores were covered by quinoa starch. Other researchers also reported the formation of a smooth coating by polysaccharides on the casein network structure (Erturk et al., 2021; Kalab et al., 1975). However, the large pores also can be seen in the structure of QF‐100 yogurt. This could be attributed to the ability of QF to block interactions between casein particles, thereby likely preventing the formation of a continuous network.

#### 3.9.1 Sensory analysis

**FIGURE 7:**
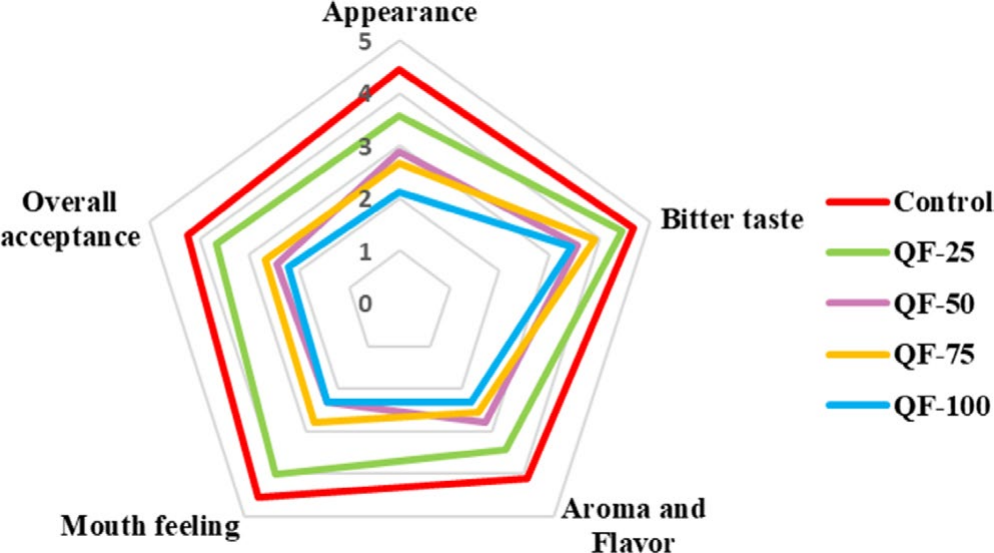
The effect of substitution of QF for SMP on the sensory properties of concentrated yogurt. Samples: Control = concentrated yogurt without QF; QF‐25, QF‐50, QF‐75, and QF‐100 = concentrated yogurt with QF substituted for SMP at levels of 25%, 50%, 75%, and 100% (w/w), respectively. Values with different letters are significantly different (*p* < .05)

**FIGURE 8:**
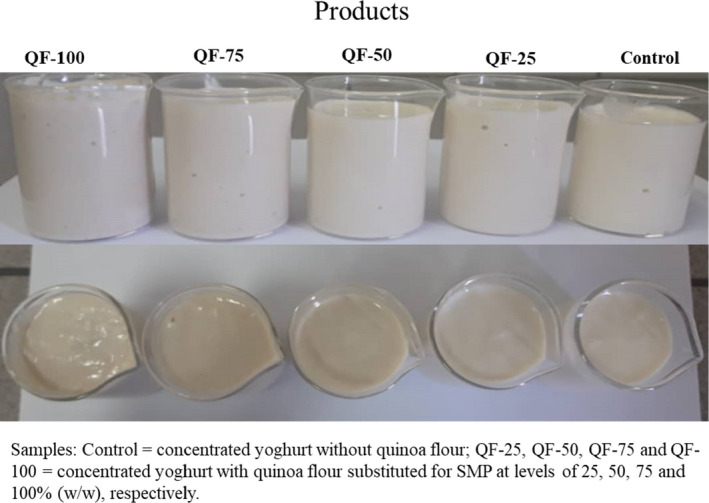
Visual observations of the control and QF‐contained yogurts

The effect of substitution of QF for SMP on the sensory qualities of concentrated yogurt is shown in Figure 7. The results showed a significant decrease in appearance acceptance with substitution beyond 25% (w/w) of QF addition to the formulation. This may be due to the creating graininess appearance and decrease in the brightness of the samples compared to control sample, which is also confirmed by the color change results and visual observation of the product (Figure 8). The bitter taste analysis revealed that the amount of bitterness in all substitution was not significant. This was probably due to the saponins removal from quinoa seeds and by the fermentation process (James, 2009; Lai et al., 2013). The substitution beyond 50% (w/w) of QF for SMP significantly reduced the aroma and flavor acceptance of the samples. The substitution beyond 25% (w/w) of QF for SMP led to a significant decrease in the mouth feeling of yogurts. Quinoa products have a sandy mouth feeling (Väkeväinen et al., 2020). This may be due to the presence of insoluble fibers and starch in QF. Eventually, the QF‐50, QF‐75, and QF‐100 samples had the lowest overall acceptance compared to the control sample. However, the taste modification of yogurts containing QF may improve the acceptance of final product in substitution of beyond 25% (w/w) QF for SMP.

## 4 CONCLUSIONS

This study showed that substitution of 25% (w/w) of QF for SMP had no statistically significant difference in chemical composition, firmness, adhesiveness, apparent viscosity, and sensory qualities with control sample while consistency was improved. Furthermore, substitution of 100% of QF improved the physicochemical properties (i.e., firmness, consistency, adhesiveness, viscosity, and carbohydrate content) compared to the control sample and led to significantly shorter fermentation times. SMP leads to an increase in the lactose content of the milk‐based yogurt and subsequently a weaker texture. Quinoa seeds are rich in essential amino acids with high nutritional value and also QF has excellent techno‐functional properties; thus, QF may be an interesting raw material for concentrating the milk‐based yogurt at substitution level of 25% (w/w).

## CONFLICT OF INTERESTS

There are no conflicts of interest to declare.

## ETHICAL APPROVAL STATEMENT

The authors declare that this study did not involve human or animal subjects, and human and animal testing was unnecessary in this study.

## Data Availability

The data that support the findings of this study are available from the corresponding author, upon reasonable request.
